# Cognitive training-induced short-term functional and long-term structural plastic change is related to gains in global cognition in healthy older adults: a pilot study

**DOI:** 10.3389/fnagi.2015.00014

**Published:** 2015-03-09

**Authors:** Amit Lampit, Harry Hallock, Chao Suo, Sharon L. Naismith, Michael Valenzuela

**Affiliations:** ^1^Regenerative Neuroscience Group, Brain and Mind Research Institute, University of SydneySydney, NSW, Australia; ^2^Monash Clinical and Imaging Neuroscience, School of Psychological Science, Monash UniversityMelbourne, VIC, Australia; ^3^Healthy Brain Ageing Program, Brain and Mind Research Institute, University of SydneySydney, NSW, Australia

**Keywords:** cognitive training, healthy older adults, magnetic resonance imaging, post-central gyrus, posterior cingulate, hippocampus

## Abstract

Computerized cognitive training (CCT) is a safe and inexpensive intervention to enhance cognitive performance in the elderly. However, the neural underpinning of CCT-induced effects and the timecourse by which such neural changes occur are unknown. Here, we report on results from a pilot study of healthy older adults who underwent three 1-h weekly sessions of either multidomain CCT program (*n* = 7) or an active control intervention (*n* = 5) over 12 weeks. Multimodal magnetic resonance imaging (MRI) scans and cognitive assessments were performed at baseline and after 9 and 36 h of training. Voxel-based structural analysis revealed a significant Group × Time interaction in the right post-central gyrus indicating increased gray matter density in the CCT group compared to active control at both follow-ups. Across the entire sample, there were significant positive correlations between changes in the post-central gyrus and change in global cognition after 36 h of training. A *post-hoc* vertex-based analysis found a significant between-group difference in rate of thickness change between baseline and post-training in the left fusiform gyrus, as well as a large cluster in the right parietal lobe covering the supramarginal and post-central gyri. Resting-state functional connectivity between the posterior cingulate and the superior frontal gyrus, and between the right hippocampus and the superior temporal gyrus significantly differed between the two groups after 9 h of training and correlated with cognitive change post-training. No significant interactions were found for any of the spectroscopy and diffusion tensor imaging data. Though preliminary, our results suggest that functional change may precede structural and cognitive change, and that about one-half of the structural change occurs within the first 9 h of training. Future studies are required to determine the role of these brain changes in the mechanisms underlying CCT-induced cognitive effects.

## Introduction

Cognitive training (CT), defined as structured practice on specific and standardized tasks targeting specific cognitive skills or neural systems (Clare et al., 2003; Gates and Valenzuela, 2010; Keshavan et al., 2014) is often suggested as a safe, scalable and relatively inexpensive means to deliver targeted cognitive challenges. A rapidly growing body of evidence supports the efficacy of CT on neuropsychological outcomes in the healthy aged (Reijnders et al., 2013; Lampit et al., 2014a), and has been influential in reforming longstanding attitudes about the inevitability of degeneration and decline in late life. However, the neurobiological adaptations that underlie CT-induced cognitive changes are only beginning to be understood (Vinogradov et al., 2012; Valkanova et al., 2014), and a solid neurobiological theory for CT effectiveness has yet to emerge. As the field gradually expands from questions of mere efficacy into mechanisms of action and optimizing practice parameters (Jaeggi et al., 2011), neuroimaging data may help to understand links between cognitive change and measures of structural and functional brain adaptation.

From a practical perspective, neuroimaging may help facilitate clinical implementation of CT by filling three major knowledge gaps. First, it can examine the extent to which CT can affect known biomarkers of brain aging (Belleville and Bherer, 2012). Second, it can help identify “training responsive brains,” that is, multivariate neuroimaging patterns at baseline that can distinguish between those individuals that will have a robust clinical response to training from those who will not, or predict the magnitude of CT-induced cognitive change (e.g., Engvig et al., 2012a). Third, better understanding of short- and long-term CT-induced neurobiological changes can lead to development of neuroimaging markers for dosing, titration and maintenance of benefits in order to guide intervention design. Such markers could potentially be more reliable than repeated neuropsychological assessments, as they are not biased by practice effects and are sensitive to longitudinal aging-related brain changes (Lustig et al., 2009).

Most of neuroimaging investigations to date tended to focus on single-domain CT paradigms, i.e., training programs that target, and typically transfer to, specific cognitive domains and related neural processes. Indeed, this approach has led to highly informative results in areas such as mnemonic training (Valenzuela et al., 2003; Belleville et al., 2011; Engvig et al., 2012a, 2010), working memory training (Dahlin et al., 2008; Brehmer et al., 2011) and spatial navigation training (Lövdén et al., 2012; Wenger et al., 2012). Whilst convenient for research purposes, the extent to which results from neuroimaging investigations of single-domain CT can inform clinical practice may be limited. This is mainly because aging is associated with simultaneous decline in most cognitive skills (Salthouse, 2012; Singh-Manoux et al., 2012), and training one cognitive domain seldom translates into others (Ball et al., 2002). Therefore, multidomain CT, i.e., programs that target a wider range of cognitive skills and multiple neural processes—may be a more suitable approach to delay or prevent aging-related cognitive decline, but their neural effects have been seldom investigated.

Neuroimaging investigations of CT could arguably maximize their clinical relevance by incorporating three design elements. First, provide a multidomain CT program that targets the main loci of cognitive aging, typically memory, speed, attention and executive functions. Second, cognitive outcomes should be expressed in terms of global cognition (GC), a composite cognitive measure that is arguably the single most robust marker of functional decline (Wilson et al., 2012), and is gaining importance as a primary endpoint in both pharmacological (Kozauer and Katz, 2013; Sperling et al., 2014) and non-pharmacological trials in non-demented older adults. Third, because various neurobiological changes might be involved, it would be beneficial to explore structural, functional and metabolic changes, and establish effect sizes across modalities. Studies using magnetic resonance imaging (MRI) techniques to investigate the effects of multidomain CT in the aged are sparse, but there is evidence of increased left hippocampal activation in people with mild cognitive impairment (Rosen et al., 2011), as well as increased volume in the corpus callosum and changes in white-matter plasticity in younger and older adults (Lövdén et al., 2010). The current body of evidence from various CT paradigm and MRI modalities (structural, functional, diffusion tensor imaging and MR spectroscopy) was recently systematically reviewed (Valkanova et al., 2014). Yet, comparisons across MRI modalities have been seldom reported, and to the best of our knowledge, no study has linked CT-induced changes in GC to possible neural correlates. Moreover, the overwhelming majority of studies so far have included only pre- and post-training timepoints, thereby missing the opportunity to explore the temporal dynamics of CT-induced neurobiological effects.

To that end, we have recently reported the results of the Timecourse Trial, a randomized active-controlled trial of a multidomain computerized cognitive training (CCT) program in 77 older adults at risk for dementia (Lampit et al., 2014b). In short, we found a significant improvement in GC in the CCT group over and beyond an active control intervention after 9 h of training, with moderate net effect sizes (NES = 0.33), that grew after 27 additional hours (NES = 0.49). The effects waned after training cessation, but residual effects were still noted 3 and 12 months post-training (NES = 0.30 and 0.21, respectively). This was the first trial to show a reliable CCT-induced effect on GC in healthy older adults, following two previous trials that did not observe such transfer (Nouchi et al., 2012; Barnes et al., 2013). Of note, these two trials used home-based, self-administered CCT interventions, whilst ours was delivered in a group-based and supervised manner, which was recently identified as a key predictor of CCT outcomes in healthy older adults (Lampit et al., 2014a).

As part of the Timecourse Trial we designed and conducted a pilot neuroimaging investigation in a subset of participants. Specifically, we aimed to (1) pilot test multimodal neuroimaging to assess effect sizes of training-induced neurobiological changes across modalities, (2) assess the temporal dynamics of training-induced neurobiological changes, and (3) compare effect sizes and examine relationships between neuroimaging and cognitive findings. All procedures and analyses were conducted as predetermined, with an emphasis on using the most recent data acquisition, processing and analysis tools.

## Materials and methods

### Study design and participants

This study was conducted using a subsample of participants from the Timecourse Trial, using the same recruitment, eligibility criteria, randomization, interventions and assessment methods. The Timecourse Trial (Lampit et al., 2014b) was a randomized active-controlled trial of group-based multidomain CCT in healthy older adults. After baseline assessment, eligible participants received a total dose of 36 1-h sessions over 12 weeks of either CCT or an active control intervention. The CCT intervention entailed exercises of memory, attention, response speed, executive functions and language, based on the COGPACK cognitive training suit (Marker Software, Heidelberg, Germany). The identical COGPACK training regimen can be obtained for inspection and replication purposes from http://rng.org.au/wp-content/uploads/2013/08/Lampit-et-al-CCT-Sessions.zip. None of training programs resembled the transfer measures (see Supplementary Material). The active control (AC) intervention was developed to control for non-specific effects inherent to supervised CCT. Participants viewed seven short National Geographic videos per session on a computer and answered multiple-choice questions immediately after each presentation. The trial was approved by the Human Research Ethics Committee at the University of New South Wales, Sydney, Australia, and prospectively registered with anzctr.org.au (identifier ACTRN12611000702910).

The primary cognitive outcome was GC, defined as a composite of memory, information processing speed, and executive functions. Cognitive assessments were performed at baseline, after 3 weeks and 3 months of training (9 and 36 sessions, respectively). Memory and information processing speed scores were obtained from the Mindstreams battery (Dwolatzky et al., 2003). Executive function score composite was defined as the average of Mindstreams Stroop Interference test and CANTAB Stockings of Cambridge problems solved in minimum moves score. GC score was obtained by averaging these three standardized scores.

Eligible participants were older adults (aged ≥ 65 years) who were fluent in English, physically able to use a computer and able to attend the training center for three sessions per week. Participants were excluded for history, diagnosis or treatment for dementia, depression in last 6 months, stroke in last 12 months, major neurological and/or psychiatric disorder requiring current treatment, lack of personal informant, already undertaking a CCT program or current alcohol abuse. Further exclusion criteria included Mini Mental State Examination (MMSE) ≤23, Informant Questionnaire on Cognitive Decline in the Elderly (IQCODE) >3.3, or Geriatric Depression Scale (15-item GDS) ≥8. Of note, none of the participants had a history of stroke. After giving their consent to participate in the trial, participants in whom MRI was not contraindicated (e.g., no metallic implants) were offered to participate in the imaging subsample in addition to the main trial. Neither consent nor refusal to participate in the imaging subsample affected inclusion, randomization, intervention or cognitive assessments, and cognitive data from participants in the imaging subsample was combined with those of the other participants. Inclusion in the subsample was determined after randomization to the main trial based on a 2:1 allocation with *N* = 18.

### Data acquisition

Multimodal MRI scans were performed on three occasions, at baseline, after nine training sessions (FU1) and after 36 sessions (FU2), using a 3.0-Tesla General Electric scanner at the Brain and Mind Research Institute, University Of Sydney. Each scan took 45–50 min to complete and included: (1) Structural (sMRI): T1-weighted whole brain scan (sequence: T1GR; TR/TE 7.1/2.7 ms; slice thickness 1 mm without gaps; field of view 256 × 256; resolution 1 × 1 mm; (2) Resting-state fMRI: T2^*^ echo-planar BOLD sequence (T2^*^EP/RG; TR/TE 2000/30; slice thickness 4.5 mm without gaps; 200 volumes, 6.5 min), eyes closed; 3) Proton Magnetic Resonance Spectroscopy (1H-MRS): in left hippocampus (20 mm M/L, 15 mm D/V, 30 mm A/P, oriented along the hippocampus) and posterior cingulate gray matter (20 mm M/L, 20 mm D/V, 30 mm A/P) using the PRESS sequence (TE/TR 30/2000 ms); and 4) Diffusion Tensor Imaging (DTI): 40 directions, TR/TE 10293/55 ms, 60 slices, 2 mm^3^ isotropic.

### MRI preprocessing and statistical analysis

#### sMRI

Two structural analyses were used. First, we performed voxel-based morphometry (VBM) longitudinal processing pipeline as per our previously published protocol (Suo et al., 2012) using SPM8 (Wellcome Trust Centre for Neuroimaging, London, UK) based VBM8 (http://dbm.neuro.uni-jena.de/) toolbox, running on Matlab 2012a (MatWorks Inc., Natick, MA). Intra-subject image normalization was performed for the three timepoints (BL, FU1, FU2) combined. A longitudinal general linear model was created by SPM8, to analyze both the preprocessed structural data and resting state fMRI functional connectivity (FC) map data. The general linear model was a flexible factorial design with three factors (subject, group and time) and one interaction (Group × Time). An F-contrast was used to test the significant interaction at whole brain level. To eliminate the multiple comparison error, the results were corrected at the whole-brain cluster-level using a false discovery rate (FDR) procedure at the *p* < 0.05 level. Finally, FC or gray matter density data from significant clusters were extracted to illustrate the direction of changes and then correlate with changes in GC scores in the imaging subsample.

Secondly, an analysis of cortical thickness was performed using the longitudinal pipeline of FreeSurfer (http://surfer.nmr.mgh.harvard.edu/). It creates a within-subject template through a variety of steps including image registration, normalization, skull-stripping, segmentation of gray and white matter, and delineation between the inner gray-white matter and outer pial surface. The resulting surface maps where then used to assess the longitudinal training effect, at both a whole-brain (QDEC) and regional level (parcellation), with changes in cortical thickness indicative of structural neuroplasticity (Reuter et al., 2012). Vertex-based analysis was conducted on the outputs from the FreeSurfer longitudinal pipeline, using the FreeSurfer QDEC toolbox. After 20 mm smoothing, three measures of cortical thickness change were used to assess for plasticity, namely: (1) annualized rate of change (±) in mm/year; (2) symmetrized percent change (SPC) with respect to the temporal average; and (3) percentage change with respect to baseline (PCL). In the post-processing model, SPC and PCL were set as dependent variable, while group was defined as the main factor. Multiple comparisons were corrected using two methods, namely whole-brain vertex based false detection rate (FDR) correction, and exploratory small-volume based FDR correction.

#### fMRI

**Figure 1:**
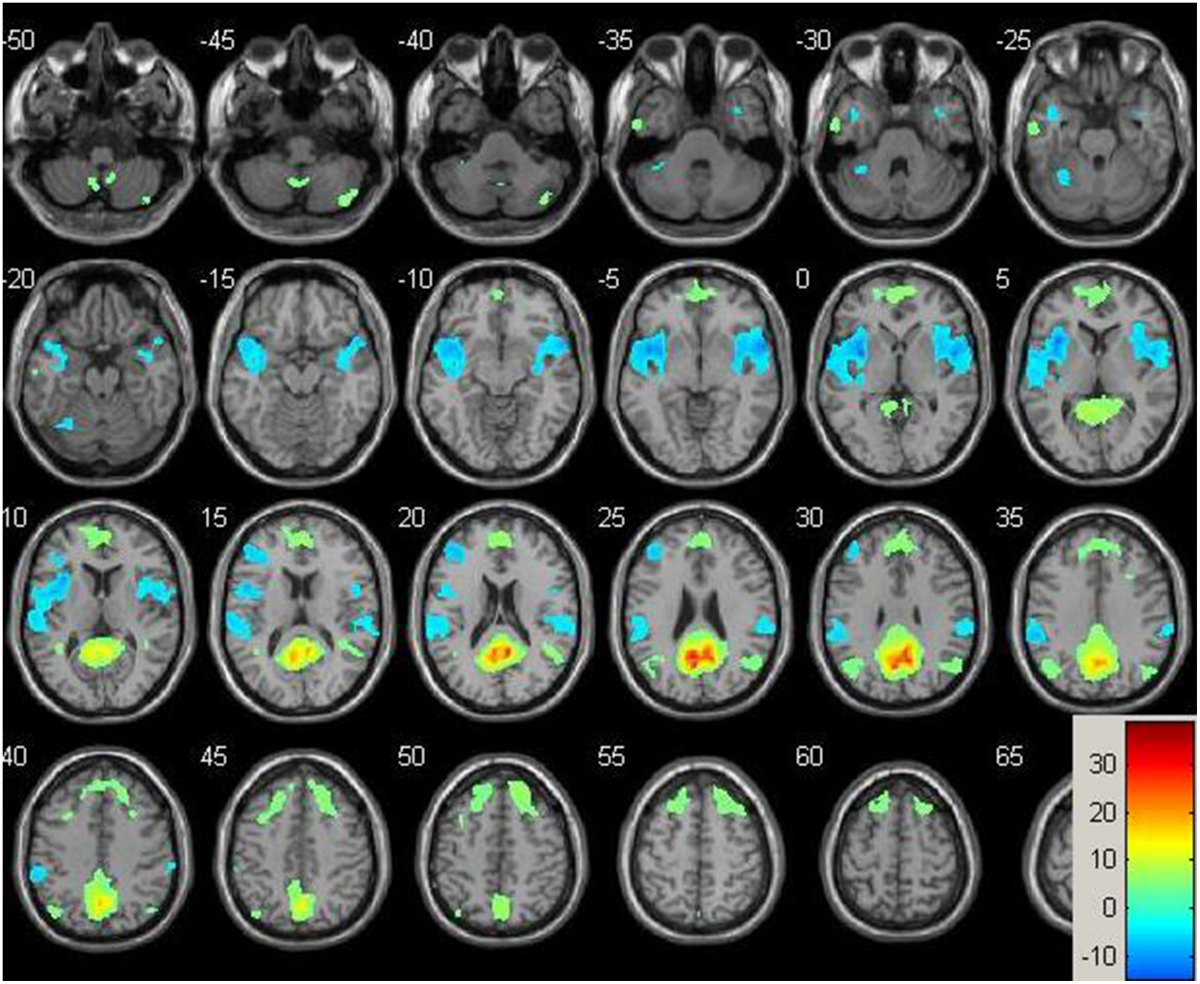
**Baseline whole-brain functional connectivity map for the posterior cingulate seed across the whole sample (*p* = 0.001, cluster size threshold >130) on a standardized single T1 template**. Hot areas represent voxels positively correlated with the seed and cool areas negative correlations.

**Figure 2:**
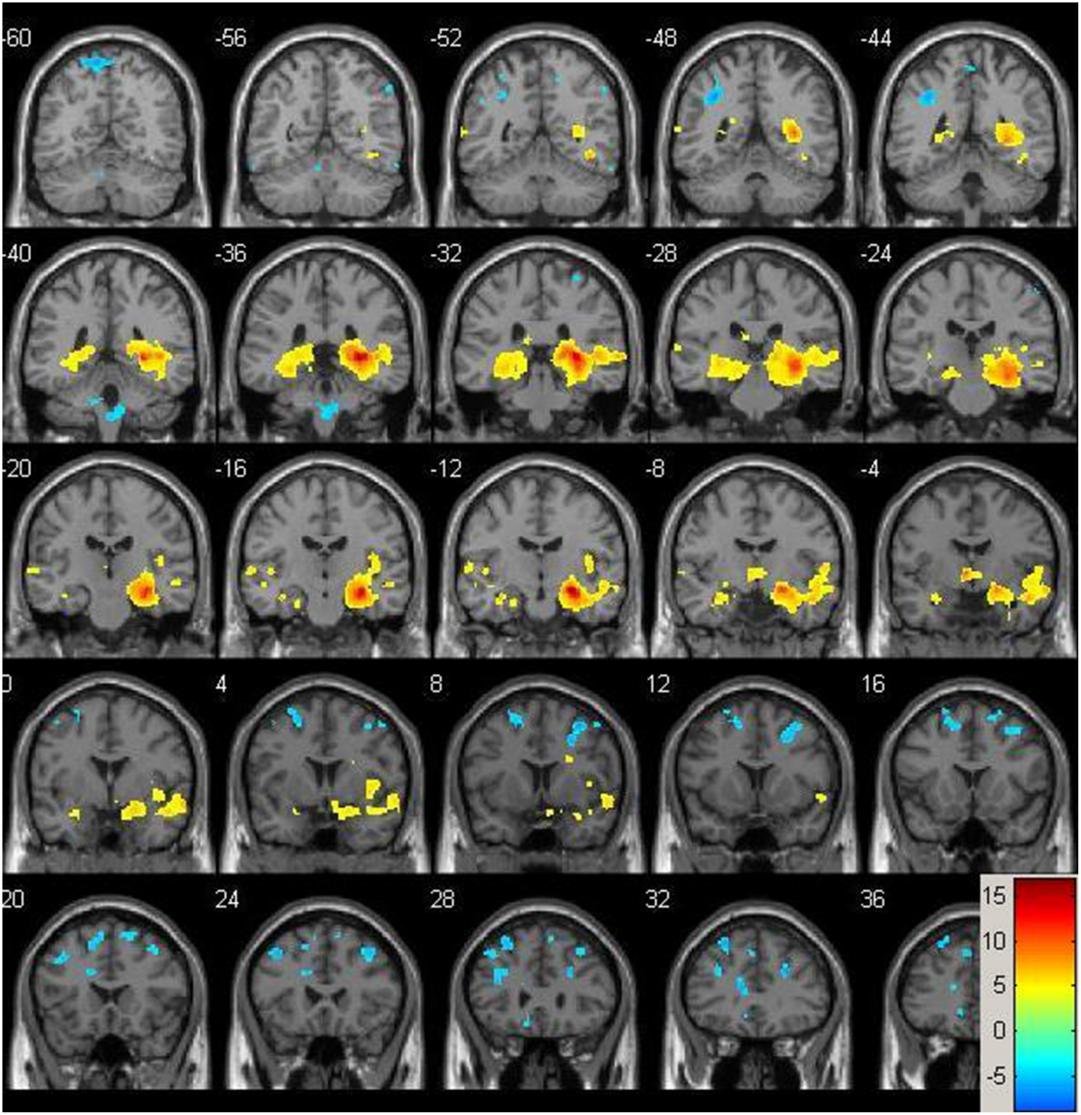
**Baseline whole-brain functional connectivity map for the right hippocampus seed across the whole sample (*p* = 0.001, cluster size threshold >130) on a standardized single T1 template**. Hot areas represent voxels positively correlated with the seed and cool areas negative correlations.

Resting-state fMRI data were preprocessed using the Data Processing Assistant for Resting State fMRI (DPARSF) toolbox of the SPM8 (Chao-Gan and Yu-Feng, 2010), including discarding the first 10 volume of each scan, motion correction, nuisance covariates regression (white matter, whole brain, CSF signal as well as 6 co-registration factors), normalizing to standard MNI space, re-sizing to 2 × 2 × 2 mm^3^ voxel, smoothing using 8 mm kernel, detrending and extracting the low frequency signal by a band-pass filter (0.01~0.08 Hz). The Resting State fMRI Data Analysis Toolkit (REST, www.restfmri.net) was used to generate pre-specified seed-wise FC maps of the hippocampus and the posterior cingulate, defined by Automated Anatomical Labeling (AAL) template (Tzourio-Mazoyer et al., 2002) (see Figures 1, 2 for baseline FC maps for the whole sample of the posterior cingulate and right hippocampus, respectively). The posterior cingulate seed was chosen for its role in the default mode network (Fransson and Marrelec, 2008), and the hippocampus seed was chosen for its role in memory and responsiveness to CCT (Rosen et al., 2011).

#### 1H-MRS

We followed our previously published protocols for MRS processing of the five main metabolite signals (Valenzuela et al., 2003; Ross et al., 2005a,b) namely N-acetylaspartate (NAA), Creatine (Cr), Cholines (Cho) Myo-inositol (mI), and Glutamate+Glutamine (Glx). Residual water signal was first removed by Hankel Lanczos Squares Singular Value Decomposition filter implemented on jMRUI. The spectra were then aligned by setting NAA peak to 2.02 ppm and baseline correction performed (using 150 data points for mean). Finally, we used the AMARES function to quantify the amplitudes of the five metabolite peaks and calculated relative amplitudes using Creatine as reference peak.

#### DTI

Voxelwise statistical analysis of the FA data was carried out using TBSS (Tract-Based Spatial Statistics, Smith et al., 2006), part of FSL (Smith et al., 2004). TBSS projects all subjects' FA data onto a mean FA tract skeleton, before applying voxelwise cross-subject statistics.

#### MRS and DTI analyses

Individual metabolic values and FA maps were analyzed using linear mixed-modeling repeated-measures analyses, with Group and Time as main factors and the interaction of interest (Group × Time) on SPSS 20. All Group × Time interactions tested changes from baseline to FU1 or change from baseline to FU2.

## Results

### Participants

**Figure 3:**
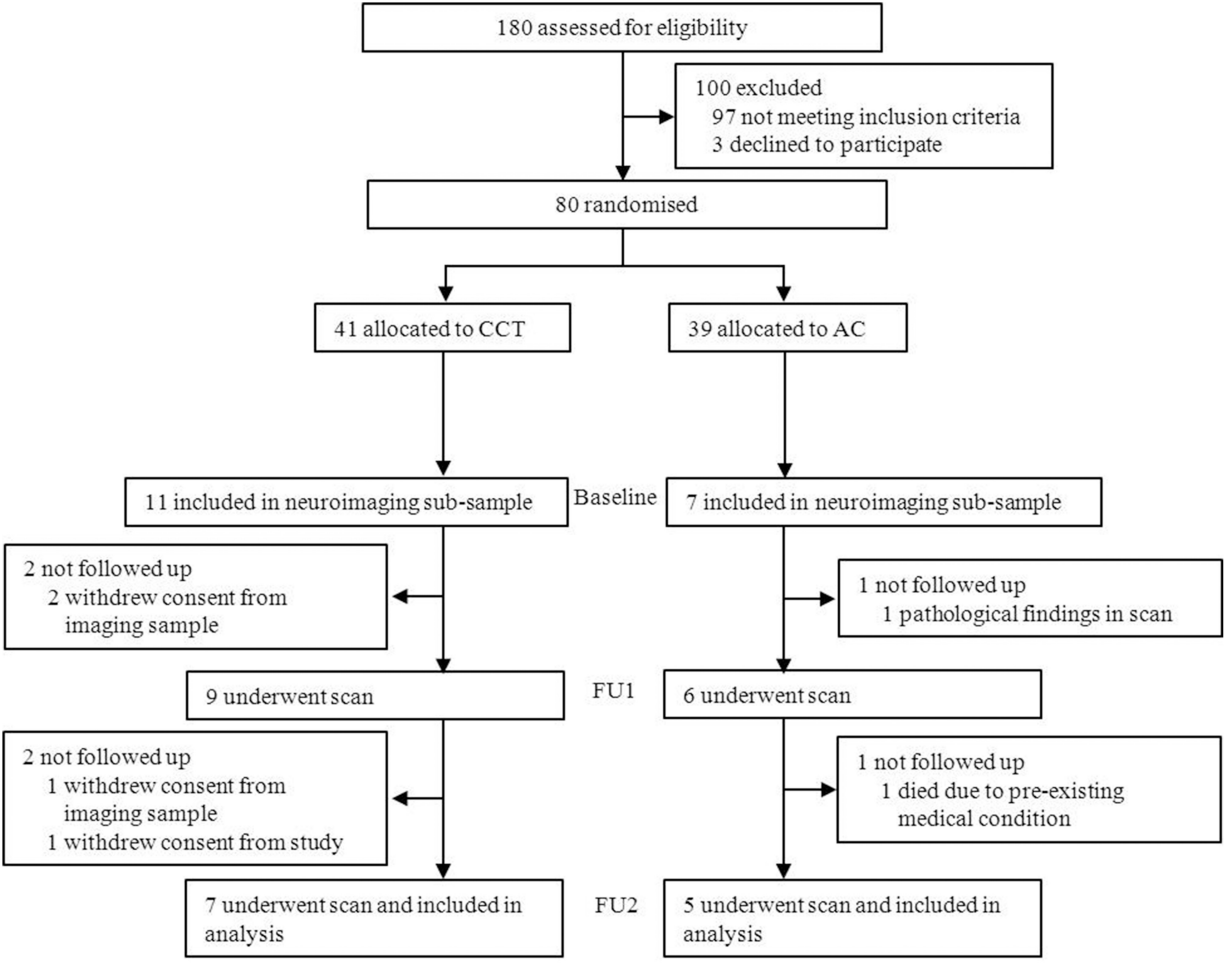
**Study design and participant flow**.

Eighteen subjects were initially included in the imaging subsample. Two withdrew consent from the imaging subsample between baseline and FU1. Two additional subjects withdrew consent and one passed away between FU1 and FU2, and one subject was excluded from the trial following pathological findings in his baseline scan (see Figure 3). A total of 12 subjects (CCT: six females, one male; AC: five males, see Table 1) were therefore analyzed on a per-protocol completion basis, using three scans per subject.

**Table 1:** **Baseline characteristics**.

	**AC (***n = 5***)**	**CCT (***n = 7***)**	***P*-value**
Age (years)	70.2 (6.7)	72.3 (8)	0.646
Male sex, No. (%)	5 (100)	1 (14)	0.003
NART-r (SD) pFSIQ^*^	110.4 (5.8)	117.4 (7.7)	0.098
MMSE (SD)	28.4 (1.6)	29 (1.1)	0.477
GDS (15-item) (SD)	1 (1.2)	0.6 (0.1)	0.424

*Data are n (%) or mean (SD). NART, national adult reading test (revised); MMSE, mini mental state examination; IQCODE, informant questionnaire on cognitive decline in the elderly; GDS, geriatric depression scale*.

* *IQ-equivalent*.

### Effects on global cognition

Repeated-measures ANOVA revealed an overall significant Group × Time interaction favoring CCT across the 3-month trial (*F* = 7.833, *p* = 0.003). The net effect size (Cohen's *d*) on GC (compared to baseline) was *d* = 0.94 at FU1 and d = 2.18 at FU2 in the imaging subsample. Of note, effect sizes in the whole sample (*n* = 77) were *d* = 0.33 at FU1 and *d* = 0.49 at FU2 (Lampit et al., 2014b).

### VBM whole brain analysis

**Figure 4:**
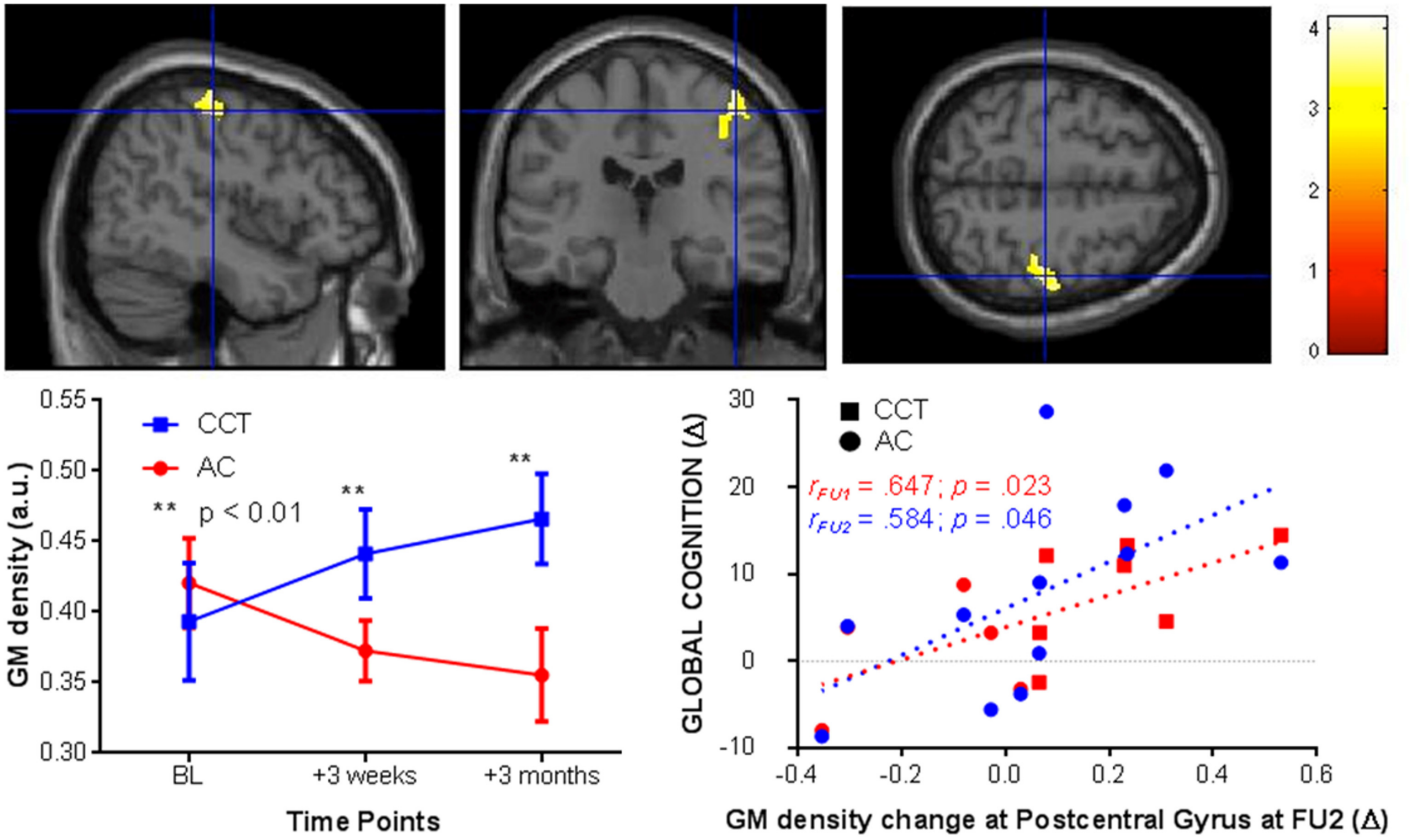
**VBM changes in the post-central gyrus at FU1 and 2 (+3 weeks and 3 months, respectively, left) and correlations between post-central gyrus change in FU2 and change in raw global cognition score at FU1 (red) and 2 (blue, right)**.

After cluster-wise FDR [clusters were threshold using *t*_(20)_ = 4.6] correction for multiple comparisons there was a significant Group × Time interaction in the right post-central gyrus (k_E_ = 1122, *p*_corr−FDR_ < 0.005 peak at [39 −25 60]). In the CCT group gray matter density increased compared to a decrease in the AC group, significant at both FU1 (z_CCT_ = 0.39; z_AC_ = −0.34) and FU2 (z_CCT_ = 0.66; z_AC_ = −0.53). See Figure 4 for a representation of these results.

We then tested for a correlation between change in gray matter density in the right post-central gyrus at FU2 and change in GC, across the entire sample. There was a significant positive correlation at both follow-ups [FU1 *r*_(1,10)_ = 0.647, *p* = 0.023; FU2 *r*_(1, 10)_ = 0.584, *p* = 0.046, see Figure 4].

### Vertex-based analysis

Whole-brain correction did not yield any suprathreshold results for any of the three measures (annualized change rate, SPC and PCL). For annualized change rate in left hemisphere, whole-brain correction yielded a significant threshold of *p* = 0.00007, whereas the vertex with minimum *p*-value was at the left fusiform gyrus (*x* = −24.3013, *y* = −43.2240, *z* = −52.2102, *p* = 0.00009). Similarly, whole-brain correction for annualized change rate in the right hemisphere yielded a significant threshold of *p* = 0.00005, whereas the vertex with minimum *p*-value across the whole sphere is at the right supramarginal/post-central gyri (*x* = 38.0839, *y* = −9.5717, *z* = 13.9501, *p* = 0.00006).

**Figure 5:**
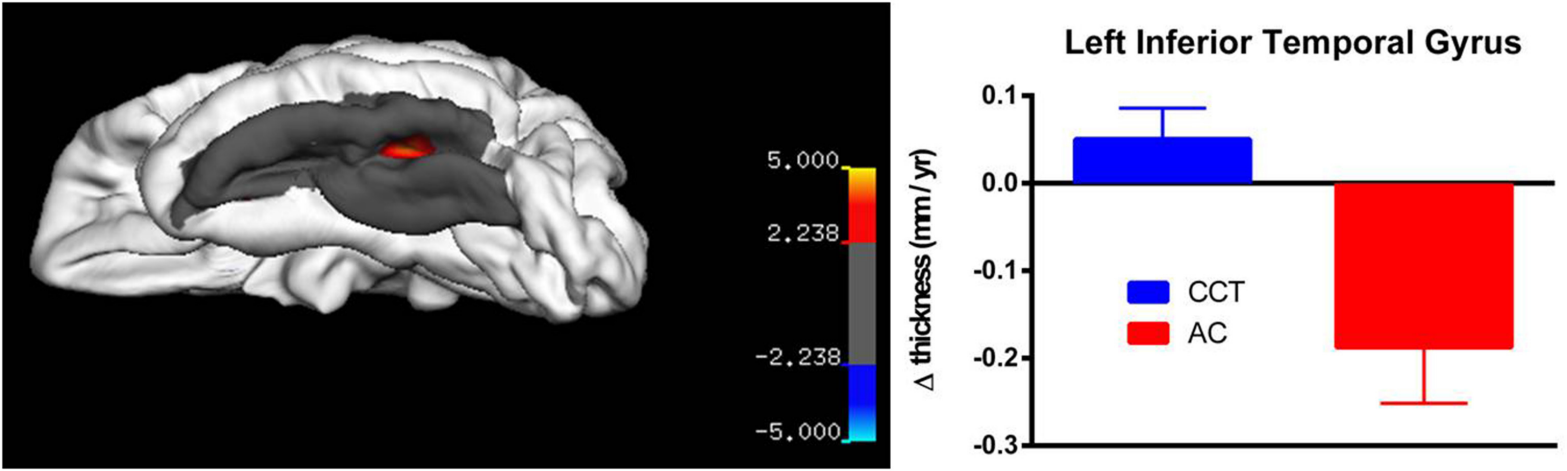
**Vertex-based analysis of the left inferior temporal gyrus**.

**Figure 6:**
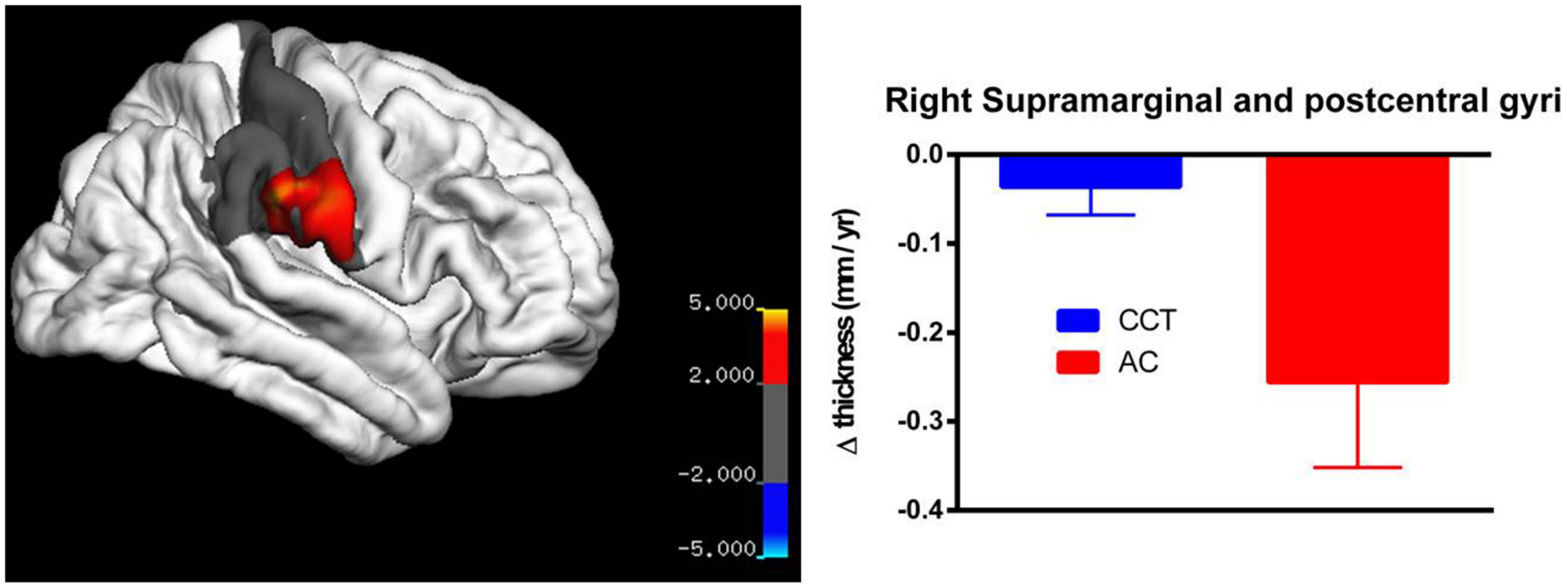
**Vertex-based analysis of the right supramarginal and post-cental gyri**.

On strictly an exploratory basis, two regions closest to threshold were identified for further testing using small volume correction, yielding a less rigid significance level (*p*_SVC−fusiform gyrus_ = 0.001; *p*_SVC−supramarginal/poscentral gyri_ = 0.006). After correction, there was a significant between-group difference in annualized rate of thickness change between baseline and FU2 in the left fusiform gyrus (*T* > 3.39, number of vertices = 9129, see Figure 5), as well as a large cluster in the right parietal lobe covering the supramarginal and post-central gyri (*T* > 2.24, number of vertices = 17288, see Figure 6). Note that for Figures 5, 6, average thickness changes was calculated after exclusion of a single outlier subject in the CCT group, as their rate of change was more than 2.5 SDs outside the group average.

### Resting-state fMRI

**Figure 7:**
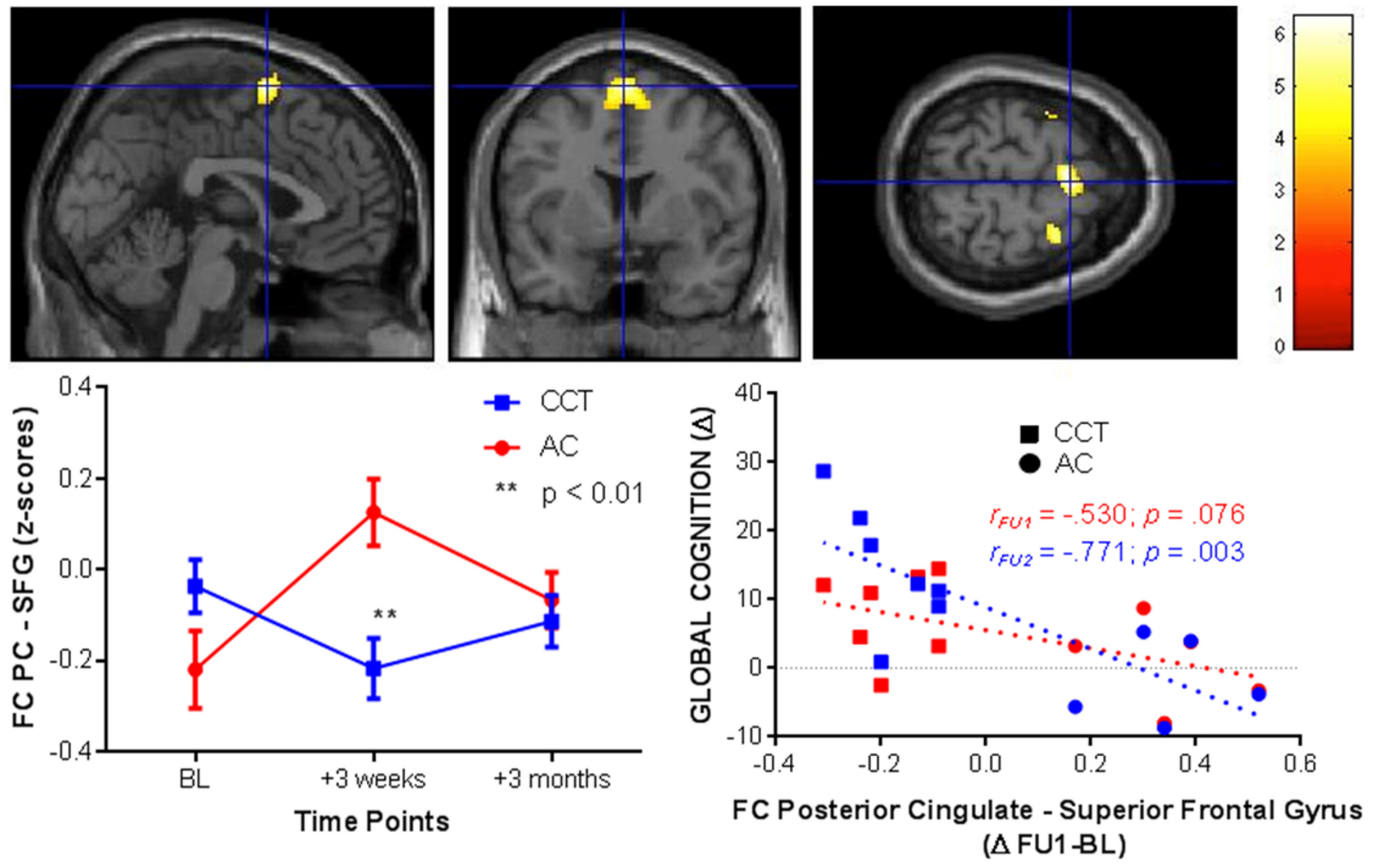
**FC changes between the posterior cingulate and superior frontal gyrus at FU1 (+3 weeks) and correlations with raw global cognition score change at the same timepoint (+3 weeks, red) and at a delayed timepoint (+3 months, blue)**.

Significant baseline functional connectivity differences between the two groups were noted for the Right Hippocampal seed (see Table 2) and the Posterior Cingulate seed (see Table 3). Compared to baseline, FC between the posterior cingulate and the right superior frontal gyrus decreased in the CCT group and increased in the AC group at FU1 (Group × Time *p* = 0.006), see Table 4. A statistically significant inverse correlation was found between the FC at FU1 and change in GC at FU2 (*r* = −0.771, *p* = 0.003), but only a trend toward significance was noted between the two deltas at FU1 (*r* = −0.530, *p* = 0.076, see Figure 7). No group differences were noted for FC change between the two regions at FU2.

**Table 2:** **Baseline whole-brain functional connectivity results for Right Hippocampus seed across the whole sample**.

**Region**	**MNI coordinates**			**Peak t**	**Peak Z**	**Cluster Size**	**P FDR _*Corr*_**
	***X***	***Y***	***Z***				
**NEGATIVE CORRELATIONS**							
Left precuneus	−8	−72	44	8.71	4.68	588	<0.001
	−12	−62	66	6.93	4.22		
	−6	−70	60	6.24	4.00		
Left inferior parietal gyrus	−34	−46	40	7.80	4.46	194	0.031
	−34	−50	56	4.43	3.29		
	−50	−52	36	4.35	3.25		
Right middle frontal gyrus	26	10	42	6.99	4.23	327	0.003
	32	8	54	6.22	3.99		
	38	24	46	5.66	3.80		
Left superior frontal gyrus	−22	6	58	8.98	3.91	280	0.006
	−12	16	52	6.55	3.76		
	−24	14	66	5.09	3.58		
**POSITIVE CORRELATIONS**							
Right hippocampus	28	−36	0	16.91	5.92	5559	<0.001
	30	−14	−16	16.57	5.88		
	32	−30	−6	11.21	5.17		
Left hippocampus	−34	−12	−20	8.36	4.60	191	0.035
	−22	−12	−24	4.61	3.37		
	−26	2	−20	4.60	3.36		
Left hippocampus	−28	−34	−8	6.62	4.12	1040	<0.001
	−22	−38	4	6.37	4.04		
	−14	−26	−10	6.04	3.93		

*Cluster size >130, p = 0.001, T = 4.0247, df = 11*.

**Table 3:** **Baseline whole-brain functional connectivity results for Posterior Cingulate seed across the whole sample**.

**Region**	**MNI coordinates**			**Peak t**	**Peak Z**	**Cluster Size**	**P FDR _*Corr*_**
	***X***	***Y***	***Z***				
**NEGATIVE CORRELATIONS**							
Left insula	−36	16	6	14.22	5.61	5356	<0.001
	−36	8	2	12.98	5.45		
	−44	8	−6	12.93	5.44		
Right superior temporal pole	60	10	−6	12.33	5.35	2725	<0.001
Right rolandic operculum	52	4	0	11.69	5.25		
Right putamen	34	−8	−8	7.78	4.45		
Left cerebellum 6	−32	−50	−26	7.16	4.28	207	0.033
Left cerebellum 6	−30	−60	−20	6.84	4.19		
Left cerebellum Crus1	−38	−46	−32	5.68	3.80		
Right supramarginal	58	−28	26	5.15	3.60	656	<0.001
	66	−32	34	5.01	3.54		
	58	−36	34	4.96	3.52		
**POSITIVE CORRELATIONS**							
Right precuneus	8	−52	22	28.56	7.24	6488	<0.001
Right precuneus	10	−58	28	34.26	7.07		
Left precuneus	−6	−54	26	30.29	6.88		
Right superior frontal	18	38	52	8.50	4.63	4151	<0.001
Right superior medial frontal gyrus	4	54	10	4.46	4.37		
Right medial orbital frontal gyrus	12	58	−2	4.45	4.36		
Right middle temporal gyrus	48	−52	20	7.90	4.48	570	<0.001
Right angular gyrus	40	−46	12	5.88	3.88		
Right angular gyrus	50	−66	28	5.52	3.74		
L cerebellum 9	−8	−60	−54	5.85	3.86	223	0.024
R cerebellum 9	8	−56	−48	4.85	3.47		
cerebellar vermis 9	0	−60	−44	4.70	3.41		
Left angular gyrus	−42	−68	36	5.52	3.75	564	<0.001
Undefined	−38	−50	12	5.42	3.71		
Undefined	−34	−58	26	5.16	3.60		

*Cluster size >130, p = 0.001, T = 4.0247, df = 11*.

**Table 4:** **Time × Group differences between FU1 and baseline**.

**Region**	**MNI Coordinates**			**Peak t**	**Peak Z**	**Cluster Size**	**P FDR _*Corr*_**
	**X**	**Y**	**Z**				
**POSTERIOR CINGULATE (ICA) SEED**							
**Positive correlation BT < CO**							
Right supplementary motor area (superior frontal gyrus)	2	6	68	6.05	4.51	310	0.006
**RIGHT HIPPOCAMPAL (AAL) SEED**							
**Positive correlation BT > CO**							
Left superior temporal gyrus	−58	−12	−4	5.60	4.29	210	0.029
Left middle temporal gyrus	−54	−12	−16	5.15	4.06		
Left inferior temporal gyrus	−50	−12	−26	4.81	3.88		

*Cluster size >50, p = 0.001, T = 3.55, df = 20. Cluster size >50, p = 0.001, T = 3.55, df = 20*.

**Figure 8:**
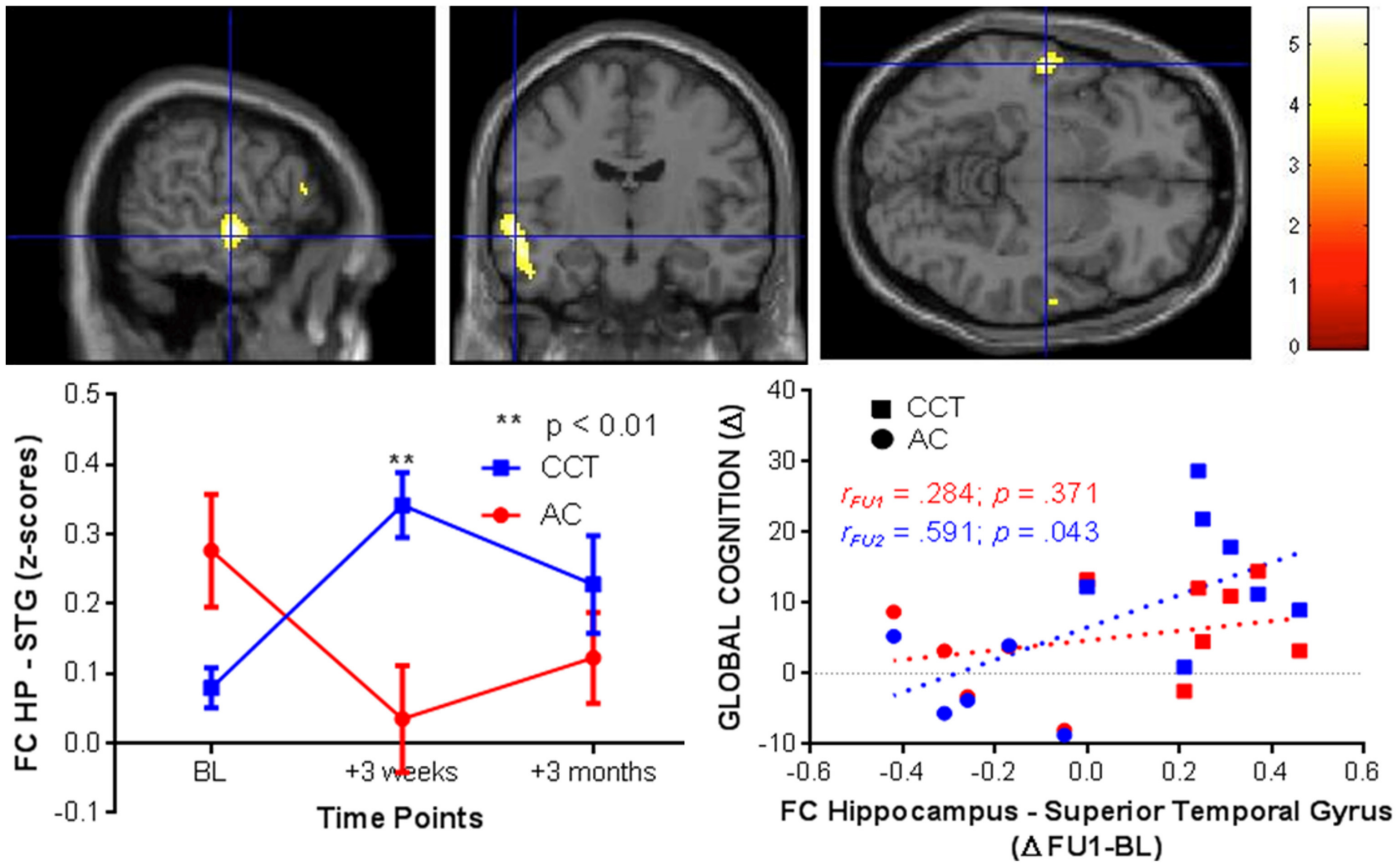
**FC changes between the right hippocampus and superior temporal gyrus at FU1 (+3 weeks) and correlations with raw global cognition score change at the same timepoint (+3 weeks, red) and at a delayed timepoint (+3 months, blue)**.

Conversely, FC between the right hippocampus and the left superior temporal gyrus increased in the CCT group and decreased in the AC group at FU1 (Group × Time *p* = 0.029), see Table 4. A statistically significant correlation was found between FC change and FU1 and GC changes at FU2 (*r* = 0.591, *p* = 0.043), but no correlation between FC change at FU1 and GC change at the same timepoint was noted (*r* = 0.284, *p* = 0.371, see Figure 8). No group differences were noted for FC between the two regions at FU2.

### Magnetic resonance spectroscopy and diffusion tensor imaging

No significant Group × Time interactions were found for any MRS metabolite (see Table 5) or whole-brain DTI analyses (Table 6).

**Table 5:** **Metabolite values (normalized to Creatine) at the three time points**.

		**Posterior Cingulate**							**Hippocampus**						
		**CCT**		**AC**		***p*-value**	**NES^a^**	**N^b^**	**CCT**		**AC**		***p*-value**	**NES^a^**	**N^b^**
		**Mean**	***SD***	**Mean**	***SD***				**Mean**	***SD***	**Mean**	***SD***			
N-acetylaspartate/Cr	Baseline	2.23	0.16	2.24	0.28	0.36	0.27	110	1.74	0.06	1.84	0.06	0.32	0.61	24
	Follow-up 1	2.14	0.07	2.25	0.29				1.74	0.11	1.72	0.05			
	Follow-up 2	2.30	0.24	2.26	0.28				1.72	0.15	1.74	0.15			
Cholines/Cr	Baseline	0.45	0.07	0.44	0.10	0.45	−0.65	22	1.06	0.05	1.12	0.10	0.49	−0.36	64
	Follow-up 1	0.43	0.03	0.46	0.12				1.09	0.05	1.13	0.17			
	Follow-up 2	0.42	0.03	0.45	0.11				1.07	0.11	1.17	0.11			
Myo-inositol/Cr	Baseline	1.01	0.14	1.11	0.18	0.10	−0.24	140	1.34	0.11	1.42	0.17	0.42	−0.42	48
	Follow-up 1	1.02	0.13	1.07	0.15				1.34	0.08	1.43	0.23			
	Follow-up 2	0.98	0.11	1.11	0.20				1.33	0.09	1.48	0.20			
Glutamate+Glutamine/Cr	Baseline	0.33	0.07	0.21	0.15	0.82	−0.99	12	0.88	0.19	0.87	0.27	0.20	−0.08	1230
	Follow-up 1	0.24	0.08	0.16	0.13				0.88	0.11	0.75	0.17			
	Follow-up 2	0.23	0.07	0.15	0.12				0.89	0.17	0.90	0.18			

*P-values refer to Group × Time effect*.

a *Net effect size (d_CCT_-d_AC_) at follow-up 2*.

b *Sample size required to detect Group × Time effect at α = 0.05 with 80% power*.

**Table 6:** **DTI measures at the three time points**.

**DTI Measures**		**CCT**		**AC**		***p*-value**	**NES^a^**
		**Mean**	***SD***	**Mean**	***SD***		
Fractional Anisotropy	Baseline	0.46565	0.02139	0.46190	0.02881	0.80	−0.03
	Follow-up 1	0.46704	0.02097	0.46622	0.02479		
	Follow-up 2	0.46590	0.02037	0.46302	0.03098		
Mean Diffusivity	Baseline	0.00077	0.00003	0.00080	0.00004	0.17	0.05
	Follow-up 1	0.00078	0.00003	0.00079	0.00004		
	Follow-up 2	0.00078	0.00003	0.00080	0.00005		
Radial Diffusivity	Baseli	0.00056	0.00004	0.00058	0.00005	0.24	0
	Follow-up 1	0.00056	0.00004	0.00057	0.00004		
	Follow-up 2	0.00056	0.00004	0.00058	0.00005		
Axial Diffusivity	Baseline	0.00120	0.00003	0.00124	0.00004	0.27	0.08
	Follow-up 1	0.00121	0.00003	0.00124	0.00004		
	Follow-up 2	0.00121	0.00003	0.00125	0.00004		

*P-values refer to Group × Time effect*.

a *Net effect size (d_CCT_-d_AC_) at follow-up 2*.

## Discussion

In healthy older adults, a CCT program effective on global cognition produced short-term functional connectivity change that preceded structural change. To the best of our knowledge, this is the first attempt to characterize the temporal dynamics of CCT-induced neural effects in older adults using multimodal imaging.

CCT effects on right post-central gyrus volume were observed in a whole-brain VBM analysis (Cohen's *d* = 1.48), and replicated in region-of-interest analysis using a more biologically plausible cortical thickness Freesurfer-based approach (*d* = 1.18). Previous neuroimaging investigations of cognitive training reported training-induced functional changes in the post-central gyrus in young (Olesen et al., 2004) and older adults (Belleville et al., 2011), as well as its possible role in predicting learning of complex video games in healthy older adults (Basak et al., 2011). Of note, neither these interventions nor the one used in our study entailed any tactile stimulation beyond normal use of a computer mouse. It is therefore possible that CCT attenuated the high rate of volume loss seen in that region in the control group. Moreover, volume change extracted from the VBM analysis correlated with positive change in global cognition, suggesting structural plasticity in this region may be therapeutically relevant to CCT in this age group. An exploratory vertex-based analysis found a between-group post-training difference in clusters around the left fusiform and inferior temporal gyri. However, a similar Group × Time difference was not found in the voxel-based sMRI analysis, and so the biological plausibility of this finding is uncertain.

Interestingly, more than a half of the structural change in the CCT group was observed after merely 3 weeks of training (FU1), while the following 9 weeks induced more moderate volume change. This finding is in line with previous studies showing that CT-induced functional (Kühn et al., 2013) and structural change (Chapman et al., 2015) tend to occur in a substantially faster rate early in the training period and tend to plateau over time. A similar pattern of diminishing return over time was also found in our larger (*n* = 77) study of the timecourse of CCT-induced GC outcomes (Lampit et al., 2014b), but not in the imaging subsample. Taken combined, these findings stress once more the importance of using multiple timepoints in CT trials.

Training-induced differences on posterior cingulate–superior frontal gyrus functional connectivity (FC) and the hippocampus–superior temporal gyrus FC occurred early in the course of training (between baseline and FU1), and then were absent after 9 more weeks of training. FC changes were therefore temporally and spatially different from structural changes, suggesting that these two types of imaging can quantify distinct CCT-related mechanisms. Importantly, both types of FC changes predicted subsequent cognitive change, and so may serve as a possible mechanistic explanation for CCT effect, as well as have potential as an early biomarker for titration of CCT dose or prediction of CCT response. The decrease in posterior cingulate–superior frontal gyrus in the CCT group as well as the inverse relationship between activity in this network and GC performance may be explained in light of observed increased FC between these two regions in patients with amnestic mild cognitive impairment compared to healthy aged controls (Bai et al., 2009). Clearly, however, given the small scale of this pilot study and baseline FC differences between the two groups, these findings will need replication in a larger study before their significance can be properly evaluated.

Lack of any significant Group × Time interactions on measures of DTI and MRS are surprising, as CT-induced effects on these measures have been documented previously in healthy elderly. It should be noted, however, that the three studies reporting effects on FA (Lövdén et al., 2010; Engvig et al., 2012b; Chapman et al., 2015) and the one study reporting effects on brain metabolites (Valenzuela et al., 2003) had substantially larger sample sizes than here. The fact that Group × Time interactions were found for sMRI and fMRI, despite the small sample size, but not for DTI and MRS measures, may indicate that the former are more useful in aiding clinical trials of CCT. Moreover, none of these studies included an active control group, and only one (non-randomized) study (Lövdén et al., 2010) used multidomain training. Multidomain training is likely to lead to more spatially distributed brain changes than repeated practice on essentially identical tasks over a long extended period. Table 5 provides sample size estimations for detecting Group × Time MRS differences based on our MRS data.

Replication of these neuroimaging effect size estimates is critical, especially in a fully randomized design. Subjects were randomized at the level of the whole trial, but not for this optional neuroimaging substudy, and hence their entry was influenced by a number of convenience factors, which resulted in unequal gender distribution between the two groups, a limitation that might have affected the results. It should also be noted that the correlations between GC and neuroimaging measures were performed on the whole sample, and thus may not be specific to CCT. Arguably, the most powerful outcome of this CCT imaging pilot study is therefore the ability to design future studies with precision. Such studies should also examine whether those functional and structural changes described here predict response to CCT and, of equal importance, whether they can serve as a biomarker for titration and maintenance of CCT effects.

In conclusion, neuroimaging investigations of CCT may have a role in revealing mechanisms underlying training-induced cognitive benefits, optimizing training programs and predicting response. Since changes in functional connectivity were sensitive to just 3 weeks (9 sessions) of training, this type of data may be particularly useful. Further research will be required to validate and extend upon these interesting preliminary findings.

### Conflict of interest statement

The authors declare that the research was conducted in the absence of any commercial or financial relationships that could be construed as a potential conflict of interest.
