# Supplementary material for: Cognitive training-induced short-term functional and long-term structural plastic change is related to gains in global cognition in healthy older adults: a pilot study
Source: Front Aging Neurosci. 2015 Mar 9;7:14. doi: 10.3389/fnagi.2015.00014 (PMC4353252; doi:10.3389/fnagi.2015.00014)
Supplement: Supplementary file 1 [file DataSheet1.DOCX]

***Supplementary Material***

**Cognitive training-induced short-term functional and long-term structural plastic change is related to gains in global cognition in healthy older adults**

**Amit Lampit^1^, Harry Hallock^1^, Chao Suo^1,2^, Sharon L Naismith^3^, Michael Valenzuela^1^***

^1^Regenerative Neuroscience Group, Brain and Mind Research Institute, University of Sydney, Sydney, NSW, Australia.

^2^Monash Clinical and Imaging Neuroscience, Monash Biomedical Imaging, Monash University, Melbourne, VIC, Australia

^3^Healthy Brain Ageing Program, Brain and Mind Research Institute, University of Sydney, Sydney, NSW, Australia

*** Correspondence:** Dr. Amit Lampit, Regenerative Neuroscience Group, Brain and Mind Research Institute, University of Sydney, 94 Mallett St, Camperdown, NSW, 2050, Australia. [amit.lampit@sydney.edu.au](mailto:amit.lampit@sydney.edu.au)

1. **COGPACK exercises used in the study**

**Note**: The descriptions below are based on exercise descriptions provided in COGPACK 8.1 help files. COGPACK is copyrighted, Marker Sofware, Landenburg, Germany.

**Anagrams:** A meaningful word must be made out of the letters provided. This exercise trains the use of meaningful linguistic material at word-level.

**Archive:** The trainee is given titles to pictures, and must then remember them either actively or passively.

**Ball:** The trainee must keep a ball bouncing using a horizontally movable paddle. This exercise trains visuomotor skills.

**Clock:** Set and read an analogue clock.

**Color & Labels:** *Task 1* - Colour labels are written in the colour they mean (e.g. word blue is written in blue), with one exception (e.g. word green is written in red). The wrong colour label must be clicked on. *Task 2* - A block of colours or patterns displays all but one of the selections shown in a multiple choice list. The missing one has to be found. *Task 3* - Short-term memory tasks with colours and labels.

**Comparisons:** Compare two simultaneously appearing character strings.

**Compass:** Recognize and enter compass points using on-screen compass

**Concepts:** Work out the concept/rule linking various terms. This exercise trains meaningful linguistic material at concept level.

**Connect:** Using mouse clicks, join up points according to given rules

**Eyewitness:** Trainees must recall short street scenes with random combinations of image, text, sound and movement elements. This exercise trains quick perception and passive reproduction of several simultaneous stimuli.

**Follow-up:** Continue a series of characters according to deducible rules.

**Guess** **Words:** Based on word length and definition, trainees must guess a word using the fewest number of letter clues. This exercise trains meaningful linguistic material at a relatively simple word-level.

**Labyrinths:** Using the mouse or cursor keys, trainees must sscape from randomly generated labyrinths which only have one solution and one exit.

**Logic:** *Task 1* –Formal comparison of abstract quantities. This exercise trains deductive thinking. *Task 2* – Complete a block of regularly ordered characters. Rule recognition. Like many intelligence tests. Task 3 – Logical “AND and OR” exercises. LOGIC “And and Or” is designed for learning some basic rules of logical combination.

**Mathematics:** Trainees must solve arithmetic problems, complex puzzles and problems using basic algebra and tasks which use everyday problems (percentages, sales tax).

**Memory:** Trainees are required to solve memory tasks using selectable material (e.g. text, graphics) and selectable recall options (e.g. immediate or delayed).

**New-or-Not:** Numerous items will be presented on the screen, and trainees must indicate if they have seen the item previously.

**Numbers:** Numerals expressed in roman, binary, hexadecimal form or in words from various languages must be entered in arabic-decimal numbers or vice versa

**Piece-work:** This is a simulation of an assembly line. Trainees must remove defective pieces.

**Position:** The position of 3-D bodies in space must be remembered or reproduced

**Reaction:** This exercise trains reaction rime, and requires trainees to respond to certain stimulus as quickly as possible according to given instructions

**Reading:** Trainees must memorize presented texts and then answer questions on it.

**Route:** Trainees must follow must note the route indicated on a map and then must reproduce this.

**Scales:** Scales must be brought into balance using as few as possible of the weights available.

**Search:** Trainees must search for a particular item hidden in a distracting background.

**Sequence:** A set of continuous performance tasks. Trainees must rapidly click on items based on their relationship to previous items according to a given rule.

**UFOs:** Use the mouse to catch UFOs flying in from random directions. This exercise trains hand-eye coordination.

**Who-or-What:** *Task 1* - The description of a person or a concept is given either letter by letter or as running letters. As soon as the item has been guessed, the stop button must be pressed and the answer entered. *Task 2* - Trainees must match labels to pictures.

**Wisdom:** Trainees must memorize quotes and the individuals who said them.

**Supplementary Table 1. COGPACK training schedule**

| **Session** | **Exercises used** | | | | | | | |
| --- | --- | --- | --- | --- | --- | --- | --- | --- |
| Session 1 | Reading | UFOs | Color&Labels | New-or-Not | Logic | Anagrams |  |  |
| Session 2 | Route | Sequences | Scales | Comparisons | Compass | Mathematics |  |  |
| Session 3 | Sequence | Eyewitness | Archive | Ball | Search | Position |  |  |
| Session 4 | Reading | Sequences | New-or-Not | Connect | Clock | Labyrinths |  |  |
| Session 5 | Memory | Sequences | Route | Reaction | Follow-up | Color&Labels | Anagrams | Position |
| Session 6 | Eyewitness | Scales | Comparisons | Compass | Archive | Numbers |  |  |
| Session 7 | Reading | Sequences | UFOs | Logic | Guess Words | Search | Position |  |
| Session 8 | Memory | Sequences | Connect | Clock | Route | Numbers |  |  |
| Session 9 | Eyewitness | Sequences | Color&Labels | Guess Words | Logic | Archive | Position |  |
| Session 10 | Sequence | New-or-Not | Scales | Comparisons | Compass | Mathematics |  |  |
| Session 11 | Memory | Route | Reaction | Logic | Search | Guess Words | Position |  |
| Session 12 | Eyewitness | Sequences | Archive | Connect | Labyrinths | Numbers |  |  |
| Session 13 | Reading | Sequences | New-or-Not | UFOs | Color&Labels | Guess Words | Position |  |
| Session 14 | Route | Sequences | Scales | Comparisons | Compass | Mathematics |  |  |
| Session 15 | Eyewitness | Sequences | Archive | Ball | Logic | Search | Position |  |
| Session 16 | Reading | Sequences | New-or-Not | Connect | Mathematics | Clock | Labyrinths |  |
| Session 17 | Memory | Sequences | Route | Reaction | Logic | Guess Words | Position |  |
| Session 18 | Eyewitness | Sequences | Archive | Comparisons | Compass | Mathematics |  |  |
| Session 19 | Reading | UFOs | New-or-Not | Logic | Sequences | Search | Guess Words | Position |
| Session 20 | Memory | Sequences | Connect | Clock | Labyrinths | Numbers |  |  |
| Session 21 | Sequence | Ball | Follow-up | Color&Labels | Archive | Concepts | Position |  |
| Session 22 | Piece-work | Wisdom | New-or-Not | Scales | Comparisons | Mathematics |  |  |
| Session 23 | Memory | Route | Sequence | Who-or-What | Clock | Position |  |  |
| Session 24 | Labyrinths | Eyewitness | Archive | Sequence | Numbers | Clock | Reaction |  |
| Session 25 | Wisdom | New-or-Not | Sequence | Logic | Color&Labels | Who-or-What | Position |  |
| Session 26 | Memory | Route | Scales | Comparisons | Compass |  |  |  |
| Session 27 | Eyewitness | Ball | Logic | Search | Sequences | Concepts | Archive | Position |
| Session 28 | New-or-Not | Connect | Labyrinths | Numbers | Sequences | Logic |  |  |
| Session 29 | Wisdom | Archive | Sequence | UFOs | Search | Position |  |  |
| Session 30 | Eyewitness | Route | Sequence | Scales | Color&Labels | Compass |  |  |
| Session 31 | Memory | Sequences | Follow-up | Clock | Guess Words | Numbers | Position |  |
| Session 32 | Reading | New-or-Not | Sequence | Ball | Color&Labels | Labyrinths |  |  |
| Session 33 | Memory | Route | Sequence | Logic | Connect | Mathematics |  |  |
| Session 34 | Sequence | Who-or-What | Comparisons | Reaction | Archive | Search |  |  |
| Session 35 | Wisdom | New-or-Not | Compass | Scales | Mathematics | Logic | Position |  |
| Session 36 | Sequence | Reaction | Clock | Color&Labels | Concepts | Route |  |  |
